# Identification of Novel Compound Heterozygous Variants of *MMP9* in Fetus With Metaphyseal Anadysplasia Type 2

**DOI:** 10.3389/fgene.2022.938457

**Published:** 2022-08-12

**Authors:** Lin Cheng, Fan Yang, Xinlin Chen, Jiawei Kang, Jiafu Li, Yuanzhen Zhang, Juan Liu, Jin Li, Jianhong Ma, Jie Duan

**Affiliations:** ^1^ Department of Obstetrics and Gynecology, Zhongnan Hospital of Wuhan University, Wuhan, China; ^2^ Hubei Clinical Research Center for Prenatal Diagnosis and Birth Health, Wuhan, China; ^3^ Wuhan Clinical Research Center for Reproductive Science and Birth Health, Wuhan, China; ^4^ Department of Ultrasound Imaging, Hubei Maternal and Child Health Hospital, Wuhan, China; ^5^ Department of Laboratory Medicine, Zhongnan Hospital of Wuhan University, Wuhan, China

**Keywords:** matrix metalloproteinase 9 gene, absence of fibula, short femur length, prenatal diagnosis, metaphyseal anadysplasia type 2

## Abstract

Matrix metalloproteinase 9 (MMP9) is an important member of the matrix metalloproteinase family and plays a key role in balancing extracellular matrix proteins. Studies have shown that the homozygous mutations in *MMP9* can lead to metaphyseal anadysplasia type 2 (MANDP2, OMIM#613073). The clinical phenotype of this disease is limited and there were only five reported cases of MANDP2 associated with homozygous *MMP9* mutations from three families. In this study, we described a case of a fetus with skeletal system malformation. The main clinical manifestations include the short bilateral femur, absence of right fibula, and curved ipsilateral tibia with short length. Importantly, two novel compound heterozygous variants of the *MMP9* gene (NM_004,994.3: c.151C > T and c.929del) were found through the trio whole exome sequencing and Sanger sequencing. This is the first report that identified the compound heterozygous variants of the *MMP9* gene associated with metaphyseal dysplasia type 2.

## Introduction

Matrix metalloproteinase nine gene [MMP9, Online Mendelian Inheritance in Man (OMIM) 120,361] is located on q11.1-q13.1 of chromosome 20, which contains 13 exons and 12 introns (Huhtala et al., 1990; Huhtala et al., 1991). The gene-encoded protein belongs to the matrix metalloprotein family. The main function of *MMP9* is to degrade and reshape the dynamic balance of the extracellular matrix. The study by Shinoda et al. demonstrated that the *MMP9* gene plays an essential role in bone development (Shinoda et al., 2008). The mutations of the *MMP9* gene have been reported to be linked with the cause of the metaphyseal anadysplasia type 2 (MANDP2) (Lausch et al., 2009). MANDP2 (OMIM: 613,073, also known as Maroteaux type) is a rare autosomal recessive disorder with the characteristic of short legs, short neck of femur, widening of the epiphysis, irregular epiphysis, and bent legs, which was first reported by Le Merrer M (LeMerrer and Maroteaux, 1998). Despite this fact, the studies that reported the association between the homozygous mutation of the *MMP9* gene and MANDP2 are very limited (Lausch et al., 2009; Sharony et al., 2017; Bonilla-Fornés et al., 2021). As an indication, the impact of compound heterozygous *MMP9* gene on MANDP2 was unknown previously. This work studied a Chinese fetus with MANDP2, presenting with the short femurs, absence of right fibula with short and curved right tibia, and we detected novel compound heterozygous variants in the *MMP9* gene using trio whole exome sequencing (WES) and Sanger sequencing.

## Methods

### Study Participants

The study participators included the aborted fetus (the proband), her parents, and the lineages, all of whom provided informed consent for participation in the study. The study was approved by the institutional review board at Zhongnan Hospital of Wuhan University.

### Genomic DNA Extraction

Genomic DNA was extracted from umbilical cord blood of the proband and peripheral blood of the family members separately, using a Qiagen DNA Blood Midi/Mini kit (Qiagen GmbH, Hilden, Germany, 69,506). NanoDrop spectrophotometer and agarose gel electrophoresis were employed in determining the purity and yield of DNA products.

### Copy Number Variation Sequencing

Genomic DNA was firstly fragmented. DNA libraries constructed by end filling, adapter ligation, and PCR amplification were subjected to massively parallel sequencing on the NextSeq 500 platform (Illumina, San Diego, CA). The sample sequences were screened using the hg19 genomic sequence as reference. Identified and mapped CNVs were investigated in publicly available databases, including Decipher (https://www.deciphergenomics.org), Database of Genomic Variants (DGV) (MacDonald et al., 2014), 1,000 genomes (1000G, http://www.1000genomes.org/), and OMIM (Hamosh et al., 2002), and their pathogenicity was assessed according to the guidelines outlined by the American College of Medical Genetics (ACMG) for interpretation of sequence variants. Variants were classified into five categories: pathogenic, likely pathogenic, variants of uncertain significance (VUS), likely benign, and benign.

### Whole Exome Sequencing

Genomic DNA was interrupted to 200bp around by fragmentation enzymes. The DNA fragments were end-repaired by adding an A base at the 3′ end. Followingly, the DNA fragments were ligated with barcoded sequencing adaptors and hybridized by Berry’s NanoWES Human Exome V1.0 (Berry Genomics, Beijing, China) according to the manufacturer’s standard operating procedure. The hybrid products after elution and collection were subjected to PCR (polymerase chain reaction) amplification and the purification and subsequently were ready for sequencing. Novaseq6000 platform (Illumina, San Diego, United States), with 150 bp pair-end sequencing mode, was used for sequencing the genomic DNA samples of the proband and the participated family members. Raw image files were processed using CASAVA pipeline v1.82 (Illumina, San Diego, United States) for base calling and raw data generation. The sequencing reads were aligned to the human reference genome (hg38/GRCh38) using the Burrows-Wheeler Aligner (Li and Durbin, 2009; Li and Durbin, 2010) tool with default parameters and PCR duplicates were removed by using Picard v1.57 (http://picard.sourceforge.net/). Verita Trekker^®^ Variants Detection System by Berry Genomics and the third-party software GATK (https://software.broadinstitute.org/gatk/) with default parameters was employed for detecting any variant. Variant annotation and interpretation were conducted by ANNOVAR (Wang et al., 2010) and the Enliven^®^ Variants Annotation Interpretation System authorized by Berry Genomics. The variants were classified into the same five categories mentioned earlier (Richards et al., 2015).

### Sanger Sequencing

Primer Blast was used to design the forward and reverse primers for *MMP9* variant sites (NM_004,994.3: c.151C > T and c.929del) (Table 1). The bidirectional sequencing was performed by Sanger sequencing after PCR. The sequencing results included all the target exon sequences, which are at least 30 bp flanking sequences. The purified sequencing results were bidirectionally aligned with the sequences published by Ensembl genome browser 90.

**TABLE 1:** Forward and reverse primers for *MMP9* gene variant sites.

Variant Site	Primers	Length of product
NM_004,994.3:	F: AGT​GGG​CTG​ATA​CCG​TCT​CTC​C	335bp
c.151C > T	R: GCC​CTC​AAA​GGT​TTG​GAA​TCT	
NM_004,994.3:	F: GCC​CCA​GGA​CTC​TAC​ACC​C	195bp
c.929del	R: CGG​GGC​TGA​ACC​TGG​TAG​A	

### Protein Structure Modeling

We performed the prediction with the online server, SWISS-MODEL (http://swissmodel.expasy.org/), to construct the three-dimensional structure of the *MMP9* protein.

## Results

### Clinical Phenotype

**FIGURE 1:**
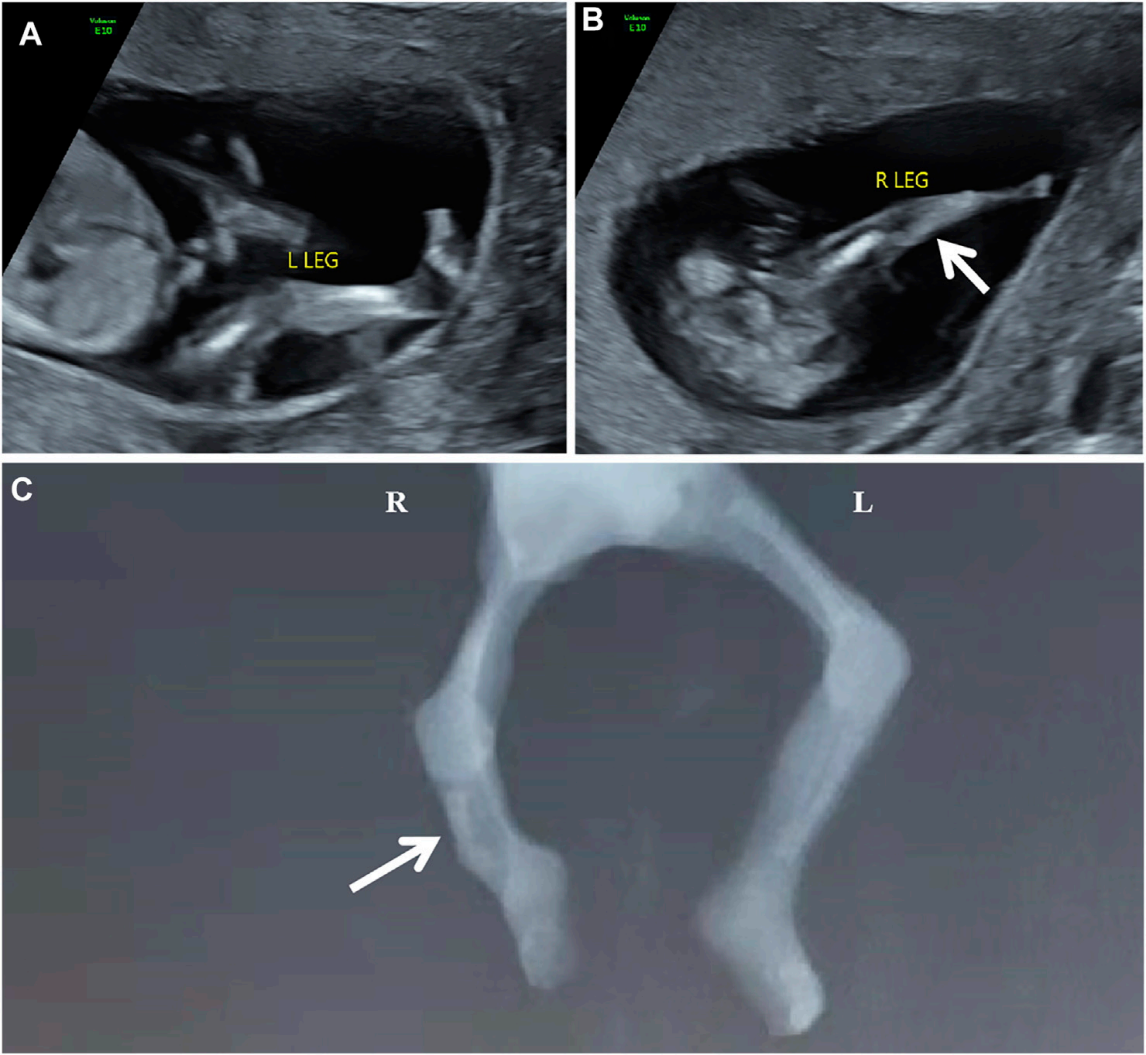
The scan images of the proband [**(A,B)** the ultrasonography at 13+6 weeks of gestation showed abnormal development of the right calf. **(C)** the X-ray of the proband showed the absence of the right fibula with the bent tibia.]

The participating couple is non-consanguineous and healthy. The pregnant woman was 31 years old. The abnormal development of the right leg with the aberrant right subclavian artery (ARSA) was detected in the fetus during 1st-trimester routine scans. One week later, a tertiary ultrasound revealed that the fetus had a single long bone echo on the right lower leg with a length of 0.6 cm, and the connection position between calf and footplate was abnormal (Figure 1AB). An ultrasound at 14+4 weeks of gestation showed a short femur (1.1 cm, −2.55 SD according to Hadlock curve) and detected a slightly curved long bone with a single echo on the right lower leg (length at 0.83 cm). Due to the fetal skeletal malformation, induced abortion was decided and performed at 15+4 weeks of gestation at the request of the couple. Meanwhile, the umbilical cord blood of proband and the peripheral blood of parents were collected for the trio whole-exome sequencing (trio WES). The copy number variant sequencing (CNV-seq) was employed to exclude the CNVs of the proband. Autopsy examination confirmed that the right fibula of the proband was absent, and the X-ray image was shown in Figure 1C.

### Laboratory Findings

**FIGURE 2:**
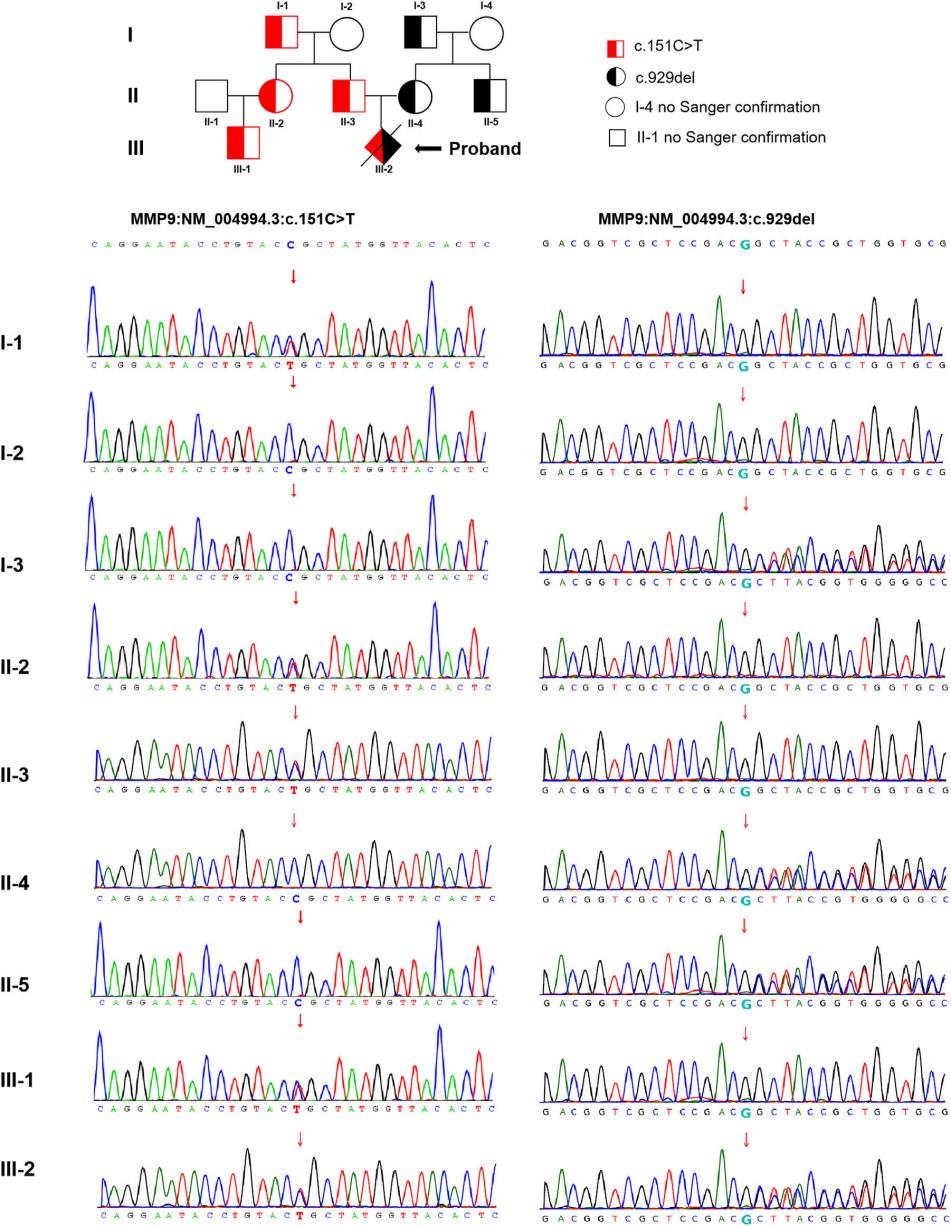
The pedigree-chart-based Sanger sequencing results of the proband (Cases I-1, II-2, II-3, and III-1 with c.151C > T, whereas cases I-3, II-4, and II-5 with c.929del.)

The trio WES detected two compound heterozygous variants in exon two and exon six of the *MMP9* gene in the proband (NM_004,994.3: c.151C > T and c.929del), with the findings that c.151C > T was identified as a paternal missense variant and c.929del was determined as a maternal frameshift variant. The analysis of *MMP9* gene variation was extended in the other family members using Sanger sequencing. These detected variants were found in multiple family members of the proband (cases I-1, II-2, II-3, and III-1 with c.151C > T, while cases I-3, II-4, and II-5 with c.929del, no blood samples obtained in cases I-4 and II-1 for personal reason.) The pedigree-chart-based Sanger sequencing results were presented in Figure 2. However, neither chromosomal aneuploidy nor pathogenic CNVs above 100bp were found using the CNV-seq.

### Protein Structure Prediction

**FIGURE 3:**
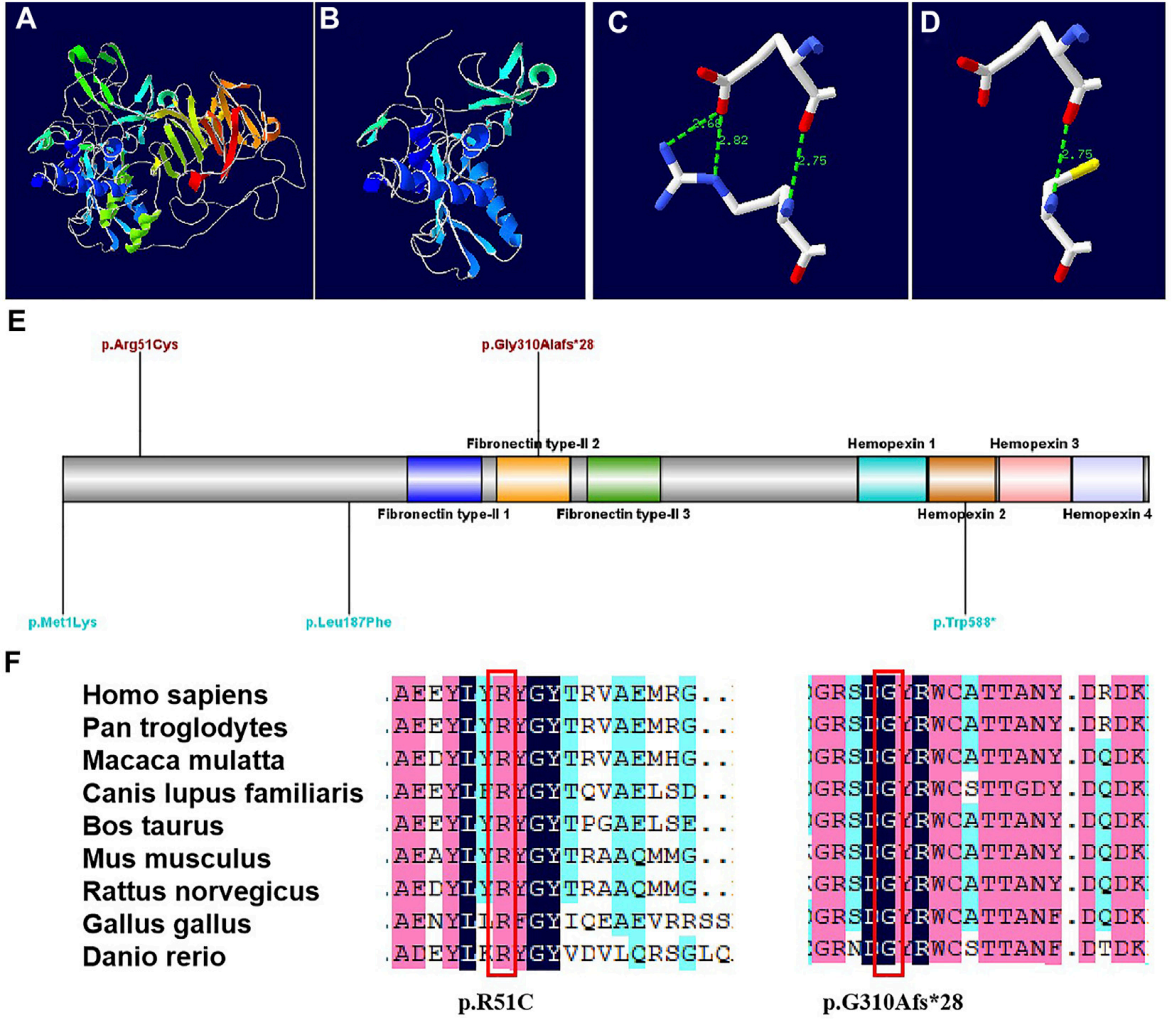
Models of the MMP9 protein, and conservation sites of the MMP9 amino acid sequences among species [**(A)**. Three-dimensional structure of normal MMP9 protein. **(B)** Three-dimensional structure of *MMP9* protein variant when a stop codon introduced at position 310.**(C)** Partial three-dimensional structure when a stop codon introduced at position 310. **(D)** Partial three-dimensional structure when Arg changed to Cys at position 51, where H bond breaks. **(E)** The variant sites previously reported are demonstrated in blue, while the variants sites reported in this case are in red. **(F)** The *MMP9* amino acid sequences were highly conserved among species.]

The protein structures of the compound heterozygous variants of the *MMP9* gene were predicted with high confidence. The predicted protein structures show a good Global Model Quality Estimation (GMQE) score of 0.67 and a Qualitative Model Energy Analysis (QMEAN) score of 0.69. The substitution of 51 Arg was identified in the structure prediction of the *MMP9* protein. The c.929del variant caused the generation of a stop codon, which can lead to a truncated protein (Figure 3AB). The *MMP9* protein prediction indicated that the p. R51C substitution could break the hydrogen bond (Figure 3CD).

## Discussion

This study reported a proband showing a short femur, unilateral absence of fibula, and short curvature of the tibia. The results of trio-WES and Sanger sequencing identified two compound heterozygous variants of the *MMP9* gene (NM_004,994.3: c.151C > T and c.929del).

The missense variant c.151C > T occurred at exon two of the *MMP9* gene on chromosomal 20, in which arginine at 51 was replaced by cysteine. To date, this variant has not been found in the 1,000 Genomes (1000G, http://www.1000genomes.org/), and the frequency of this variant in Exome Aggregation Consortium (ExAC, http://exac.broadinstitute.org/) and Genome Aggregation Database (gnomAD, https://gnomad.broadinstitute.org/) are 9.52 × 10^−5^ and 9.6 × 10^−4^, respectively (PM2). This variant was a compound heterozygous with the c.929del (PM3). Besides, this variant was predicted of the domain by InterPro software (http://www.ebi.ac.uk/interpro/), and there is no benign variation in this functional domain (PP3).

The c.929del is a frameshift variant that occurs in exon six of the *MMP9* on chromosomal 20. This frameshift variant was caused by introducing a stop codon at the following 28 amino acids, leading to the truncation of the protein by changing the open reading frame of the gene (PVS1). Nevertheless, the variant has not been reported in the 1000G, ExAC, or gnomAD databases (PM2). Uniprot analysis showed that Gly at position 310 was in the crimp domain of the *MMP9* ion channel pore, and the loss of this amino acid indicated a decreased stability or rigidity of the crimp motif.

Generally, the findings demonstrated that the discovered variants were novel. Both amino acid sequences were highly conserved among species (Figure 3F). According to the guidelines of the American College of Medical Genetics and Genomics (ACMG), the missense variant c.151C > T was classified as a “variant of uncertain significance (VUS)”, whereas the frameshift variant c.929del was classified as “likely pathogenic (LP)” (Richards et al., 2015).

There are three MANDP2 reports determined with *MMP9* gene homozygous mutation, which were shown in Table 2 and Figure 3E. In our study, we found the compound heterozygote variants of the *MMP9* gene (NM_004,994.3: c.151C > T and c.929del) through trio WES, which were recognized as novel variants. Besides the shortening of fetal lower limb long bones, the unilateral fibula absence and tibial curvature were clinical phenotypes in the case. It is well known that vascularization is crucial for transforming cartilage scaffolds into the bone during fetal skeletal development. The osteoblasts invade cartilage with blood vessels and form the bone matrix (Watson and Adams, 2018). Studies have shown that the MMP family plays an important role in bone formation and growth (Paiva and Granjeiro, 2014; Paiva and Granjeiro, 2017). Among them, *MMP9* is mainly secreted by chondrocytes, osteoclasts, and endothelial cells, and it can regulate the bioavailability of vascular endothelial growth factor A (VEGF-A). The fetal fibula deficiency in the case of our study could result from the compound heterozygous various of the *MMP9* gene, which is associated with VEGF-A reduction and impaired angiogenesis during endochondral ossification, consequently affecting the formation of the fibula. The loss of the fibula could further cause the curvature of the tibia due to the missing support from the fibula. However, the assumption requires further investigation.

**TABLE 2:** Summary of clinical features and molecular findings in MANDP2 patients caused by *MMP9* variants.

Cases	Variant	Type of Variant	Origin	Clinical findings
Two siblings (Lausch et al., 2009)	NM_004,994.3: c.21T > A	Homozygote	Pakistan	Normal stature, genu varum and metaphyseal fraying during infancy
Two siblings (Sharony et al., 2017)	NM_004,994.3: c.559C > T	Homozygote	Israel	The first one was diagnosed with shortening of the long bones during the 14.5 weeks of gestation and aborted. The second one was born with a severely reduced body length without associated finding of lethal skeletal dysplasia
One child (Bonilla-Fornés et al., 2021)	NM_004,994.3: c.1764G > A	Homozygote	Spain	Bowed legs at 19 months old. Clinical and radiological examination revealed scoliosis, genu varum and metaphyseal abnormalities. By the age of 39 months, lower limb alignment and metaphyseal features had already significantly improved, and scoliosis had disappeared
The fetus in our study	NM_004,994.3: c.151C > T and c.929del	Compound heterozygote	China	A short femur and unilateral absence of fibula with short curvature of tibia

Aberrant right subclavian artery (ARSA) is also considered in the investigation. It occurs both as a variant of normal and in association with other cardiac malformations or chromosomal abnormalities. Other studies have reported the incidence of ARSA in the second trimester as about 35% in trisomy 21 fetuses and 1.4% in chromosomally normal fetuses (Borenstein et al., 2008; Svirsky et al., 2017). A multi-center study found increased risks of chromosomal aneuploidy and pathogenic copy number variations (pCNVs) of fetal ARSA with extracardiac abnormalities (Maya et al., 2017). The presence of an isolated ARSA is benign and is not associated with chromosomal abnormalities. In our case, neither chromosomal aneuploidy nor copy number variation was found in the proband, and ARSA-related gene variant was also absent in WES and CNV-seq. Therefore, we considered ARSA to be a normal anatomical variation in this study.

## Conclusion

In summary, we have identified the novel compound heterozygous variants of the *MMP9* gene (NM_004,994.3: c.151C > T and c.929del) in the fetus with MANDP2. The compound heterozygous variants could be associated with the absence of unilateral fibula and bent tibia. However, since the c.151C > T is a VUS variant, it is necessary to research the regulation of protein structure and function by this variant, which will not only be helpful to expand the spectrum of *MMP9* gene variations and the clinical phenotype of MANDP2, but also provide guidance for prenatal diagnosis and clinical genetic consultation.

## Data Availability

The data presented in the study are deposited in the GenBank repository, accession numbers are SAMN29672838, SAMN29672839, and SAMN29672840.
